# Ubiquitin Ligase gp78 Targets Unglycosylated Prion Protein PrP for Ubiquitylation and Degradation

**DOI:** 10.1371/journal.pone.0092290

**Published:** 2014-04-08

**Authors:** Jia Shao, Vitnary Choe, Haili Cheng, Yien Che Tsai, Allan M. Weissman, Shiwen Luo, Hai Rao

**Affiliations:** 1 The First Affiliated Hospital, Nanchang University, Nanchang, Jiangxi, China; 2 Department of Molecular Medicine, the University of Texas Health Science Center, San Antonio, Texas, United States of America; 3 Laboratory of Protein Dynamics and Signaling, National Cancer Institute, Frederick, Maryland, United States of America; Deutsches Zentrum für Neurodegenerative Erkrankungen e.V., Germany

## Abstract

Prion protein PrP is a central player in several devastating neurodegenerative disorders, including mad cow disease and Creutzfeltd-Jacob disease. Conformational alteration of PrP into an aggregation-prone infectious form PrP^Sc^ can trigger pathogenic events. How levels of PrP are regulated is poorly understood. Human PrP is known to be degraded by the proteasome, but the specific proteolytic pathway responsible for PrP destruction remains elusive. Here, we demonstrate that the ubiquitin ligase gp78, known for its role in protein quality control, is critical for unglycosylated PrP ubiquitylation and degradation. Furthermore, C-terminal sequences of PrP protein are crucial for its ubiquitylation and degradation. Our study reveals the first ubiquitin ligase specifically involved in prion protein PrP degradation and PrP sequences crucial for its turnover. Our data may lead to a new avenue to control PrP level and pathogenesis.

## Introduction

Ubiquitin (Ub) is an abundant small protein best known as a molecular flag that marks proteins for destruction by the 26S proteasome in eukaryotes [1], [2]. The key specificity factors in Ub-mediated proteasomal proteolysis are Ub-protein ligases (E3s), which recognize substrates and attach chains of Ub molecules onto them with the help of several other enzymes (i.e., E1 and E2) [1], [2]. Ub-mediated proteolysis serves two major purposes: protein concentration modulation and protein quality control [2], [3], [4], [5]. Perturbations in the Ub system can lead to cancers and neurological disorders [3].

The close relationship between neurodegeneration and the Ub-proteasome system (UPS) is well documented as Ub and proteasome-positive protein aggregates have been found in various neuropathological studies [3], [6], [7]. One protein that is subject to Ub-mediated proteolysis is the prion protein PrP [8], [9], [10], [11], the scrapie form of which (PrP^Sc^) is a causative agent in transmissible spongiform encephalopathies (TSEs) or prion disorders that include fatal familial insomnia, Kuru, Creutzfeltd-Jacob disease, scrapie in sheep and mad cow disease [12], [13]. Mature PrP is a glycoprotein that is anchored to plasma membrane. PrP has been implicated in cell adhesion, axonal transport, copper homeostasis, cell signaling, and protection from apoptosis; nevertheless, the precise physiological function of PrP remains elusive [11], [12], [13]. A fascinating feature of PrP is its conformational alteration and the resulting biological consequences. PrP normally adopts a predominately α-helical structure but can be switched to a mostly β-sheet form termed PrP^Sc^, which triggers insoluble protein aggregation, clogs the proteasome, and elicits neurotoxicity [12], [13]. The PrP^Sc^ conformation can be propagated like a genetic element and transmitted like an infectious agent [12], [13], [14]. Compelling evidence supports a major role of PrP in prion maladies. The mechanism underlying the conversion of PrP's conformation remains poorly understood [12], [13], [14].

In mammals, inhibition of the proteasome leads to accumulation of prion PrP, which could be extremely toxic [11], [15], [16]. In addition, prion protein in the disease-associated conformation was found to inhibit the proteasome *in vivo* and *in vitro* [11], [17], suggesting a mechanism for PrP-induced neurodegeneration and highlighting the need to delineate the detailed functional role of the UPS in PrP destruction. Although proteasome-mediated proteolysis has been shown as one important pathway to regulate levels of PrP, how it gains access to the proteasome as a consequence of ubiquitylation remains enigmatic. Challenges in defining specific degradation route for PrP include large number of human E3s (∼600) [2] and the multiple forms of PrP (e.g,, differences in glycosylation, membrane association, or protein conformation), which may be differentially regulated [8], [9], [11], [18], [19], [20].

One Ub-mediated proteolytic route implicated in PrP regulation is endoplasmic reticulum (ER)-associated degradation (ERAD) [4], [9], [10], [15], a protein quality control system in the ER. As secretory proteins traverse ER, their folding states are checked. Malfolded proteins are selected for retrotranslocation and destruction by the proteasome in the cytosol [4], [5]. Multiple Ub ligases are employed to eliminate various aberrant ERAD substrates that are sorted based on the location of the misfolded domain (e.g. membrane, lumen, or cytosol) and the topology of the protein [4], [5]. A fraction of PrP proteins, mainly unglycosylated species, are found in the cytosol upon proteasome inhibition with the ER signal peptide removed, suggesting that PrP is regulated by ERAD [9], [10], [15]. The specific mammalian Ub ligase involved in ERAD-mediated PrP turnover was not known. We previously established the use of yeast as a model system [21], which contains protein quality control systems similar to human and has much smaller number of Ub-protein ligases [1], to study PrP degradation. We demonstrated that unglycosylated form of human PrP (ugPrP) is the preferred target of the proteasome in yeast, and further determined that the yeast Hrd1 E3 pathway, a branch of ERAD, is key to ugPrP ubiquitylation and degradation [21]. Here, we extend our findings to mammalian cells. We demonstrate that PrP interacts specifically with the Ub-protein ligase gp78, one of the mammalian orthologs of yeast Hrd1. Furthermore, unglycosylated PrP turnover is impaired in cells in which gp78 activity is compromised. Interestingly, the C-terminal structured region of PrP is pivotal for its ubiquitylation and degradation. Our study reveals key sequences crucial for PrP turnover. These results provide important stepping-stones towards understanding how PrP levels are modulated and to further unravel specific role of proteolysis in prion biology.

## Results

### E3 ligase gp78 specifically interacts with PrP

**Figure 1:**
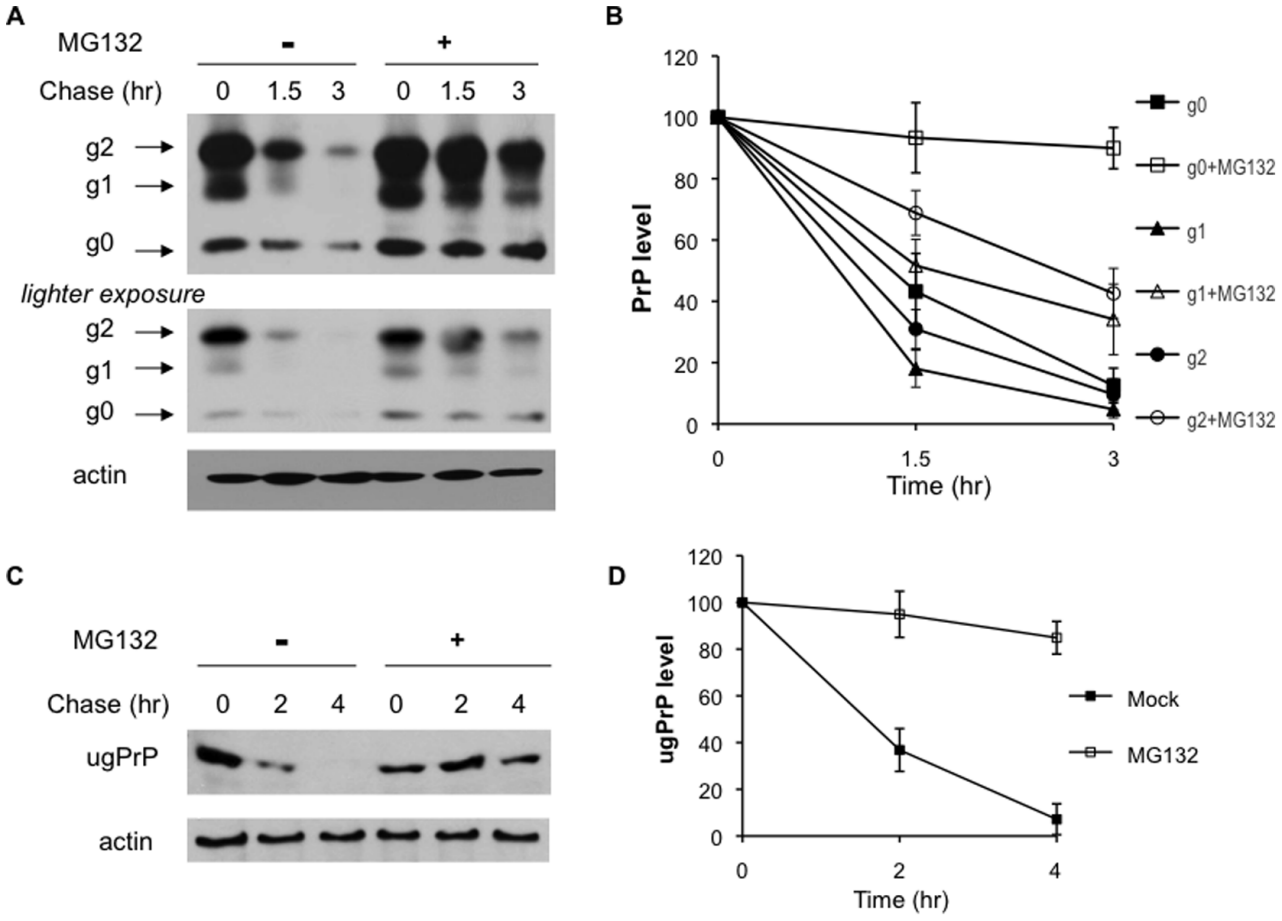
PrP degradation involves the proteasome. (**A**) PrP is degraded by the proteasome. HEK293 cells transfected with PrP were treated with or without the inhibitor of the proteasome MG132, cycloheximde was added to shut off protein synthesis and start the chase. Indicated times were taken and processed for immunoblotting with antibody recognizing PrP (3F4) or actin control. The arrows indicate PrP proteins attached with two (g2), one (g1) or no (g0) glycan. As some signals were saturated in the cycloheximide chase results presented (top panel), a lower exposure of the blot was included (middle panel). (**B**) Quantitation of the data in A. Three different glyco-forms of PrP were analyzed separately as indicated using ImageQuant software. We chose non-saturated bands for quantitation and normalized with the loading control actin. Whereas the bands for g1 species were quantified using the blot more heavily exposed (top panel), the g0 and g2 species were quantified with the lighter blot (middle panel). The experiments were done at least three times, and the average values with standard deviation are shown. (**C**) ugPrP turnover requires the proteasome. The plasmid expressing ugPrP (N181Q and N197Q) devoid of glycosylation was transfected into HEK293 cells. ugPrP stability in the presence or absence of MG132 was conducted as described in (A). (**D**) Quantitation of the data in C from 3 experiments.

The proteasome has been shown to regulate both endogenous and transfected prion protein PrP [8], [9], [10], [11], [15], [19], [22]. We first carried out the cycloheximide chase experiments to ascertain the involvement of the proteasome in human PrP turnover. We transfected plasmid encoding wild-type PrP into human embryonic kidney (HEK) 293 cells, which does not express endogenous PrP [8], [9], [11], [15], [19]. As expected [9], [11], [15], [18], [22], multiple glycoforms (i.e., di-, mono-, un-glycosylated) of PrP were detected. Consistent with earlier studies [8], [9], [10], [15], [22], in cycloheximide chase experiments all three PrP species were stabilized upon proteasome inhibition with unglycosylated PrP (g0) showing the most significant stabilization (Figure 1A and 1B). Studying the degradation of all three forms of PrP simultaneously presents challenges because of the ongoing glycosylation and de-glycosylation *in vivo* [11], [18]. For example, the disappearance of mono-glycosylated PrP (g1) over time could be due to additional glycosylation, deglycosylation, or degradation. Given the results in Figure 1A and 1B, we decided to focus on the mechanism underlying the degradation of an unglycosylated PrP (ugPrP) mutant with both residues for N-glycosylation (aa 181 and 197) mutated to Gln (to generate ugPrP). This non-glycosylatable form of PrP protein showed a striking stabilization in response to MG132, which inhibits proteasome function by cycloheximde chase (Figure 1C, D).

**Figure 2:**
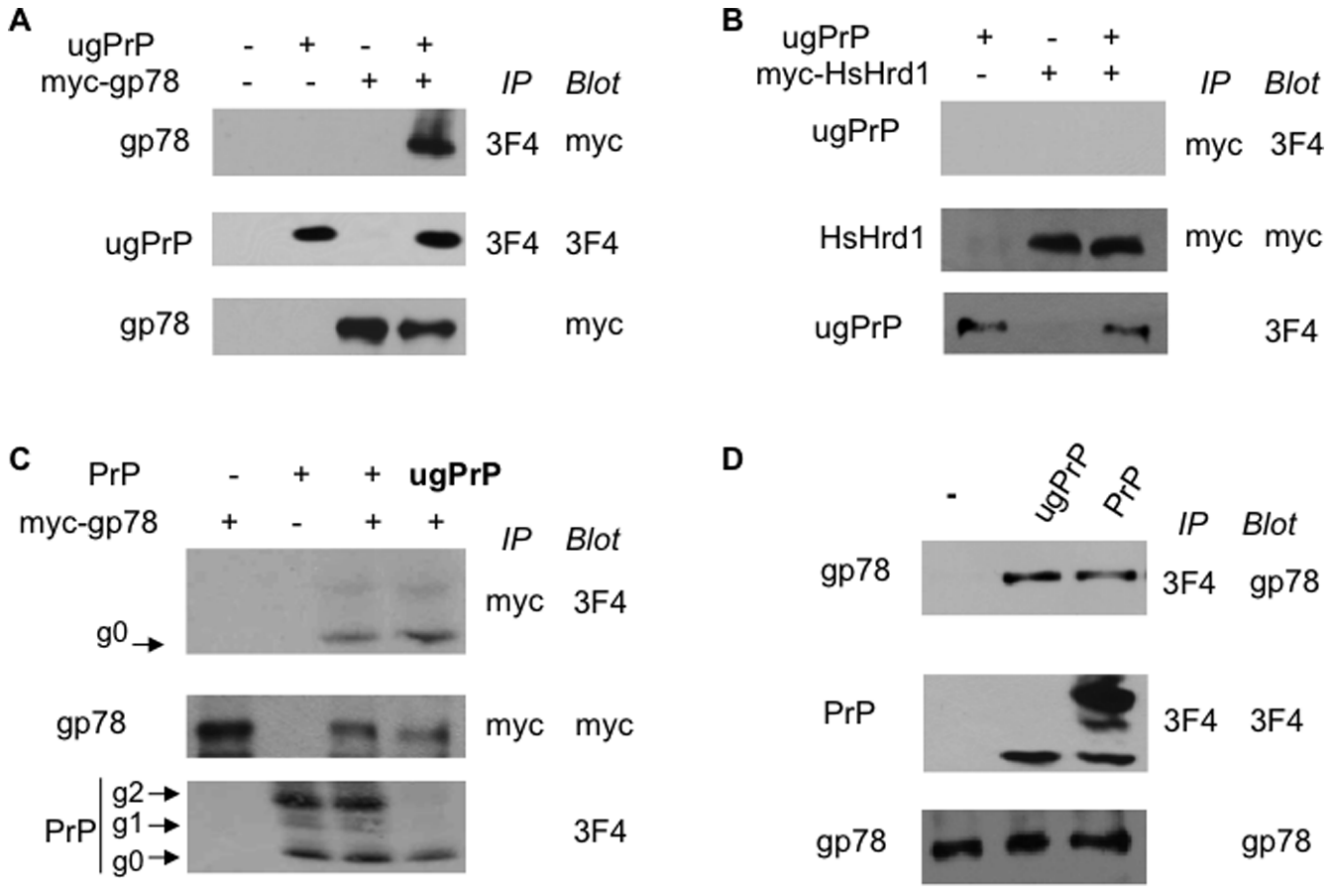
gp78 specifically interacts with PrP. (**A**) Co-immunoprecipitation analysis of interactions between gp78 and ugPrP. HEK293 cells were transfected with myc-tagged gp78 and/or ugPrP as indicated. Proteins were extracted and immunoprecipitated with beads coated with PrP antibody 3F4. Immunoprecipitates were separated on SDS-PAGE, and probed with anti-myc (top panel) or anti-PrP (middle panel). The amounts of myc-gp78 in cell extracts were evaluated and presented in bottom panel. (**B**) HsHrd1 does not bind ugPrP. Proteins were extracted from cells expressing myc-tagged HsHrd1 and ugPrP. The indicated immunoprecipitations and immunoblottings were carried out as described above in (A). (**C**) gp78 binds unglycosylated PrP preferentially. HEK293 cells were transfected with plasmids expressing myc-gp78 and wild-type PrP or ugPrP (last lane). Immunoprecipiation was carried out using anti-myc beads, and later eluted with myc peptides. Western blotting was done as described above. Only unglycosylated g0 form of PrP (lane 3, top panel), which migrated at the same position as ugPrP control (lane 4), was detected in myc-gp78 immunoprecipitation. (**D**) Endogenous gp78 interacts with PrP. HEK293 cells were transfected with the plasmid expressing wild-type PrP or ugPrP. Cell extracts were subjected to immunoprecipitation with beads coated with PrP antibody and immunoblotting with gp78 antibody.

Human ugPrP expressed in yeast is regulated by the RING-finger E3 Hrd1 [21], which has two human homologues HsHrd1/Synoviolin and gp78/RNF45 [2], [23]. We evaluated the involvement of HsHrd1 and gp78 in ugPrP turnover in mammalian cells. As Ub ligases often directly interact with their substrates, we first assessed the interaction between ugPrP and myc-tagged versions of gp78 and HsHrd1 in HEK293 cells by co-immunoprecipitation. ugPrP specifically associated with myc-gp78 but not HsHrd1 (Figure 2A, 2B and Figure S1 in File S1). We then examined whether gp78 associated with wild-type PrP proteins that bear mixed forms (Figure 2C). ugPrP was included as a control (Figure 2C, lane 4). Interestingly, gp78 specifically co-immunoprecipited unglycosylated PrP (Figure 2C, lanes 3 and 4), supporting a specific role of gp78 in unglycosylated PrP regulation. Furthermore, we found that endogenous gp78 associates with ugPrP and PrP (Figure 2D). Combined, our results suggest that gp78 may be a Ub ligase involved in PrP turnover.

### gp78 regulates the ubiquitylation and degradation of ugPrP

**Figure 3:**
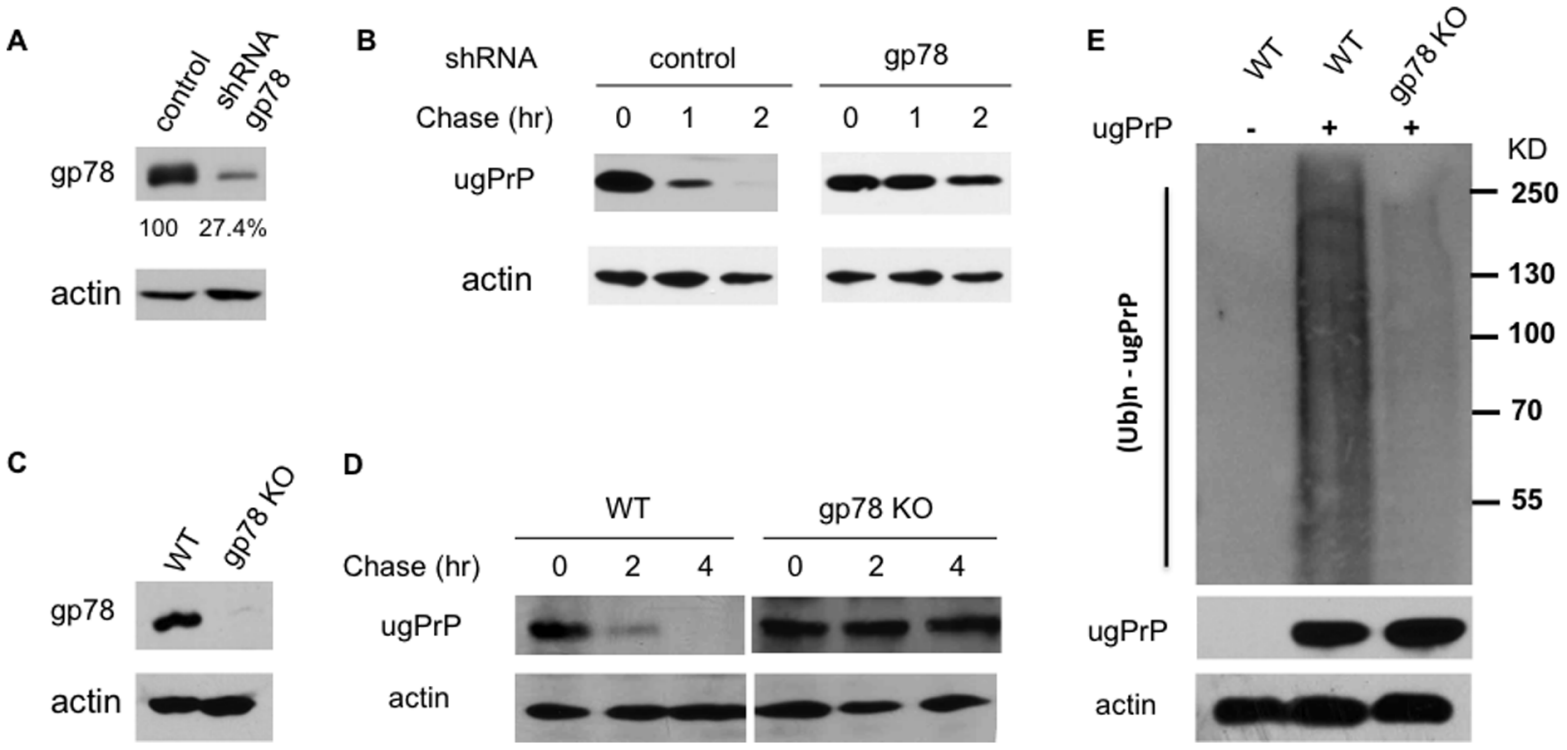
gp78 promotes ugPrP ubiquitylation and degradation. (**A**) gp78 knockdown efficiency in HEK293 cells. HEK293 cells were transfected with the gp78 shRNA or control plasmid. Cell extracts were subjected to immunoblotting analysis to determine the levels of gp78 and actin (loading control). Knockdown efficiency (27.4%) was indicated underneath the panel. (**B**) Effect of gp78 knockdown on ugPrP turnover. Degradation kinetics of ugPrP was assessed in gp78 knockdown or control HEK293 cells. Actin is shown as a loading control. Protein stability assay via cycloheximide chase was done as described in Figure 1. (**C**) gp78 deficient MEFs. gp78 expression was evaluated by western blotting in gp78^−/−^ and control MEFs. (**D**) ugPrP degradation is impaired in gp78^−/−^ MEFs. gp78^−/−^ and control MEFs were transfected with the plasmid encoding ugPrP. ugPrP turnover was evaluated by cycloheximde chase as in Figure 1. (**E**) gp78 is important for ugPrP ubiquitylation. gp78^−/−^ and control MEFs expressing ugPrP were treated with MG132 for 6 h and then lysed, and later subjected to immunoprecipitation with PrP antibody. The immunoprecipitates were analyzed by western blotting with Ub antibody and 3F4 for ugPrP (upper panels). The amount of actin in cell extracts was assessed by immunoblotting with actin antibody (bottom panel).

To directly address whether endogenous gp78 is required for ugPrP degradation, we established a stable gp78 knockdown in HEK293 cells (Figure 3A). We assessed the degradation kinetics of ugPrP in gp78 knockdown and control cells. ugPrP was rapidly degraded in control cell, but significantly stabilized in gp78 knockdown cells (Figure 3B and Figure S2 in File S1), supporting that ugPrP turnover involved gp78. Consistent with the lack of binding between ugPrP and HsHrd1 (Figure 2B), HsHrd1 knockdown did not alter ugPrP turnover (data not shown), suggesting that HsHrd1 does not play a major function in ugPrP degradation.

To ascertain the involvement of gp78 in ugPrP destruction, we transfected the plasmid bearing ugPrP to gp78^−/−^ mouse embryonic fibroblasts (MEFs) (Figure 3C). ugPrP was degraded in control MEF cells, but stabilized in gp78^−/−^ MEFs (Figure 3D). Furthermore, we assessed the requirement of gp78 for ugPrP ubiquitylation. We examined the ubiquitylation pattern of ugPrP in wild-type MEFs and gp78^−/−^ MEFs (Figure 3E). ugPrP proteins were enriched from cell extracts with PrP antibody and subsequently analyzed by western blotting with Ub antibody. Ubiquitylated ugPrP was detected as high molecular weight smears in wild-type MEFs, but markedly reduced in gp78^−/−^ MEFs (Figure 3E), suggesting that gp78 promotes ugPrP ubiquitylation. The amounts of actin in extracts were determined as controls. To our knowledge, gp78 is the first ubiquitin ligase implicated in the degradation of ugPrP protein in mammalian cells.

### C-terminal sequences are crucial for ugPrP degradation

**Figure 4:**
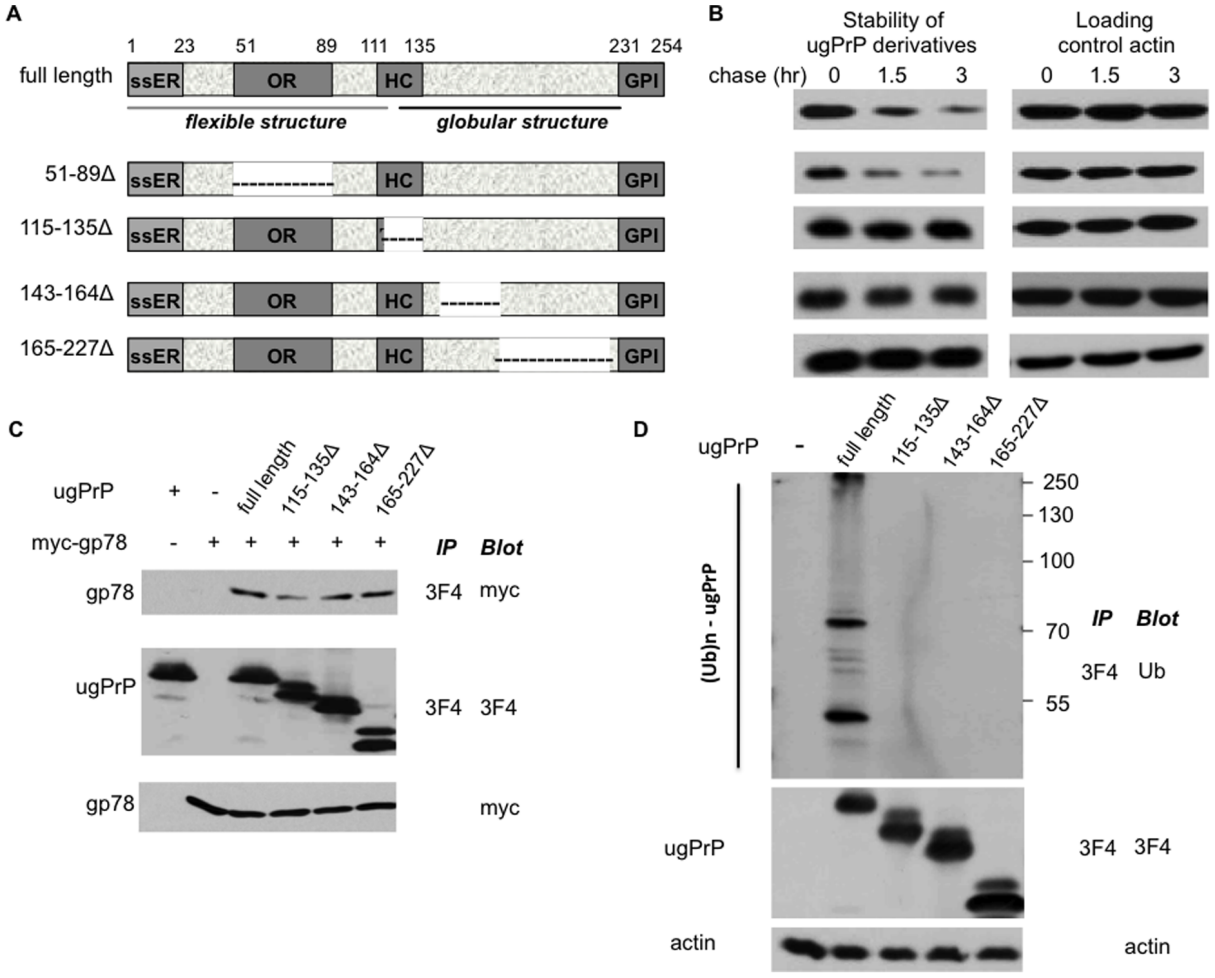
The C-terminal sequences are critical for ugPrP ubiquitylation and turnover. (**A**) Domain structure of PrP and various deletion mutants constructed. Four domains (i.e, ssER, OR, HC, GPI) are indicated. NMR analysis demonstrated disorder structure for the N-terminal segment and globular structure for the C-terminal half of PrP. The deleted portions are shown as dashed lines. (**B**) The effects of various deletions on ugPrP stability. Degradation kinetics of wild-type ugPrP and mutants (left panels) in HEK293 cells was assayed as described in Figure 1. Actin serves as the loading control (right panels). (**C**) The C-terminal deletions maintain efficient gp78-binding. The interaction between gp78 and and ugPrP derivatives was assessed as described in Figure 2A. (**D**) Deletions of C-terminal sequences impair ugPrP ubiquitylation. The ubiquitylation pattern of ugPrP derivatives in HEK293 cells was determined as described in Figure 3E after MG132 treatment.

To further understand PrP degradation, we sought to identify sequence elements in ugPrP critical for its destruction through deletional analysis. PrP contains four domains: an ER-targeting signal sequence (ssER) that is cleaved off in the ER, an octameric repeat region (OR) critical to formation of PrP^Sc^, a hydrophobic region that is inhibitory to prion PrP^Sc^ biogenesis, and the glycophosphatidyl inositol (GPI) anchoring sequence (Figure 4A) [12], [13], [18], [24]. Structural analysis indicates that normal PrP contains a flexible N-terminal region (amino acids 23–124) and a folded C-terminal domain with three α-helices (amino acids 144–156, 172–193, 200–227), which is key to prion formation and pathogenesis [13], [24]. We constructed internal deletions in ugPrP and then examined their stability *in vivo* (Figure 4A). In these constructs, both the ER-targeting signal and the GPI anchoring sequences, which are central to PrP's proper localization and processing in the ER, remain intact. Interestingly, whereas disruption of the OR region did not alter ugPrP degradation, deletions encompassing the hydrophobic region or α-helices led to significant ugPrP stabilization (Figure 4B and Figure S3 in File S1), indicating that these C-terminal sequences and likely the structural integrity of PrP are critical for ugPrP instability.

We then evaluated the specific molecular defect(s) that the C-terminal deletions may bring about. The ability to bind gp78 E3 and also the ubiquitylation profile of these ugPrP derivatives were examined. Interestingly, these deletions maintained efficient gp78-binding but led to drastically reduced ugPrP ubiquitylation (Figure 4C and 4D).

## Discussion

PrP protein plays a central role in prion diseases as it is the major component in purified infectious material [12], [13], [24]. PrP also controls many key features of prion diseases such as incubation time, susceptibility, and species barriers. Mice lacking PrP are immune to prion infection. Determining the regulation of cellular PrP holds a key to understanding what may go awry in prion diseases [11], [13], [22]. PrP is known to be degraded by the proteasome [9], [11], [15], [22]. Moreover, PrP^Sc^ impairs the proteasome *in vivo* and *in vitro*, suggesting a mechanism for PrP-induced neurodegeneration [11], [17]. This also begs the question of how PrP gains entry to the proteasome, which often requires Ub as the ticket. To delineate the detailed functional relationship between PrP and the proteasome-mediated proteolysis, it is crucial to identify key cis- and trans-determinants involved in PrP turnover. Since the globular domain of PrP is required for PrP turnover (Figure 4), it will be interesting to evaluate whether the altered conformation in this region of PrP^Sc^ spares the scrapie form of the protein from degradation. It is also possible that some pathogenic mutations in the C-terminal region of PrP may affect PrP turnover, which could facilitate PrP^Sc^ biogenesis [8], [11], [13], [19].

Our results reveal gp78 as the first Ub ligase specifically required for PrP degradation in mammalian cells. The Ub ligase gp78 belongs to a family of E3s containing a RING finger motif [2], [23]. In cooperation with the E2 enzyme Ube2g2, gp78, an integral ER membrane protein, participates in ERAD [5], [23]. PrP is routed through the ER to the plasma membrane to fulfill its normal, albeit unknown function(s) [12], [13], [18]. Like other secretory proteins, the folding state of PrP is actively monitored in the ER. Terminally misfolded secretory proteins are disposed through ERAD to prevent toxification by the accumulation of aberrant proteins. More specifically, misfolded proteins are returned to the cytosol via a poorly defined retrotranslocation mechanism, tagged with Ub by E3s (e.g., gp78), and subsequently destroyed by the proteasome in the cytosol [4], [5]. Consistent with the known involvement of gp78 in ERAD, a fraction of PrP with the ER signal sequence removed in the ER was shown to be retrotranslocated to the cytosol for proteasomal degradation, suggesting that PrP is an ERAD substrate [9], [10], [15], [16], [19], [21]. A sign of PrP maturation is deemed as N-glycosylation [12], [13], [18]. In line with this notion, in mammalian cells, MG132-induced PrP accumulation mostly involves non-glycosylated PrP [8], [9], [10], [15], [22]. Importantly, gp78 preferentially recognizes the unglycosylated form of PrP (Figure 2C) and is critical for ugPrP ubiquitylation and degradation (Figure 3).

To delineate cis-elements key to ugPrP destruction, the importance of various recognizable domains in PrP protein for its turnover was analyzed (Figure 4). Interestingly, sequence alterations in the folded C-terminal region of PrP lead to compromised ugPrP ubiquitylation and degradation without affecting gp78-binding. E3 ligase catalyzed ubiquitylation requires at least three elements in the substrate: the E3-recognition domain, Ub-attachment site(s) (e.g., mainly internal Lys residues but could be Cys, Ser or N-terminal α-amino group), and the accessibility of Ub-attachment site [1], [2]. Lysine residues are not deleted in two C-terminal mutants (i.e., 115–135Δ, 143–164Δ). Given apparent ubiquitylation and degradation defects of ugPrP C-terminal mutants (Figure 4B and 4D), structural alteration(s) induced by the deletions likely affect the accessibility of ubiquitylation site, a poorly understood issue in the Ub field. Further structural and functional analysis of PrP ubiquitylation may reveal how E3s select specific residue(s) for Ub conjugation.

Multiple forms of PrP exist and are likely regulated by distinct pathways [8], [9], [11], [17], [18], [19], [20]. Both glycosylated and unglycosylated PrPs are present in protein aggregates, unglycosylated ugPrP, by either *in vitro* synthesis or mutating its glycosylation sites, can efficiently induce *in vivo* aggregate formation, suggesting the unglycosylated form is critical for prion formation [12], [13], [18], [24]. Mature glyco-PrP proteins reach the plasma membrane and are later subjected to endocytosis and destroyed by the lysosome [11], [13], [18], [24]. Identification of the gp78 pathway in PrP regulation will help elucidate the functional cooperation between two different proteolytic systems (i.e., the proteasome and lysosome) in prion biology.

We took a detour in identifying key components involved in PrP turnover. Given the large number of Ub ligases in human, we employed yeast *S. cerevisiae* as a model organism towards this goal [21]. With its facile genetics, well-characterized genomics, and a vast array of biochemical assays and tools, yeast offers a powerful system that accelerates the pace of discovery. We previously found that ugPrP is degraded by ERAD in yeast [21], consistent with the implication of ERAD in PrP degradation in mammals [9], [13], [19]. Our results here indicated that PrP is regulated by a homologous pathway in mammalian cells, further validating the use of yeast as a model organism to study PrP regulation.

The link between the proteasome and PrP appears to be physiologically significant since PrP^Sc^ impairs proteasomal activities, which are essential to cell growth and survival [11], [13], [17]. Importantly, it was shown that tuning down PrP expression can reverse the progression of disease even after its onset [25], [26]. Our data not only provide new insights regarding the mechanism governing PrP regulation, but also lay the foundation for unraveling the specific roles of Ub-mediated proteolysis in prion biogenesis and pathogenesis.

## Materials and Methods

### Cell lines and Plasmids

HEK293 cells were cultured using Dulbecco's modified Eagle's medium (DMEM) containing 10% fetal bovine serum and penicillin/streptomycin. Construction of mice bearing a heterozygous mutant allele for gp78 has been described [23]. Heterozygous mice (gp78^+/−^) mice were crossed with C57BL/6 for at least 10 generations for this study. Primary mouse embryonic fibroblasts (MEFs) were prepared from somites of E12.5 embryos from mating gp78^+/−^×gp78^+/−^ as described [23]. Animal protocol (ASP 10-207) was approved by NCI Animal Care and Use Committee. All media reagents were purchased from Cell-gro. Cells were incubated at 37°C with 5% CO_2_. Cells were transfected with X-tremeGENE 9 DNA (Roche) with over 60% transfection efficiency. The stable knockdown cells were obtained using the pLKO lentiviral system by Open Biosystems (Huntsville, AL). Cells were infected with viral supernatants containing the gp78 shRNA plasmid (Open Biosystems) or control plasmid. gp78 knockdowns were isolated using selective reagent (puromycin 2 µg/ml) and ascertained by western blotting using gp78 antibody, a gift from Dr. Yihong Ye (NIH).

The plasmid pCEP4β-PrP expressing human PrP was a kind gift from Dr. Andrea LeBlanc [10]. The plasmids expressing PrP deletions (Δ51–89, Δ115–135, Δ143–164, Δ165–227) and glycosylation mutant (ugPrP; N181Q and N197Q) were generated via the Quick Change Mutagenesis kit (Stratagene, Carlsbad). The plasmids expressing myc-tagged HsHrd1 and myc-tagged gp78 were obtained from Drs Billy Tsai and Yihong Ye.

Antibodies and beads conjugated with andibodies – Antibodies against PrP (3F4) and actin were purchased from Chemicon (Temecula, CA), and gp78 antibody was obtained from Yihong Ye (NIH). Antibodies against Ub and myc (9E10), and myc conjugated beads were obtained from Covance (Berkley, CA), rProtein A beads were purchased from GE Healthcare Life Sciences.

### PrP degradation assay

Cells transfected with the plasmid expressing PrP or ugPrP were treated with 100 µg/ml cycloheximide to inhibit protein synthesis at ∼48 hours post transfection. Samples were taken at the indicated time points and lysed in RIPA buffer (50 mM Tris-HCl pH 7.5, 150 mM NaCl, 1% Sodium deoxycholate, 0.5% SDS, 0.5% NP-40, 1 mM EDTA) supplemented with protease inhibitors. Samples were processed for western blotting with PrP antibody 3F4 (Chemicon; Temecula, CA). The stable protein actin was employed as the loading control to ensure that equal amounts of extracts were used. After the protein bands were detected by the ECL system and scanned, their densities were analyzed by ImageQuant software.

### Co-immunoprecipitation/immunoblotting assay

HEK293 cells were co-transfected with plasmids expressing PrP derivatives and/or myc-tagged gp78 or HsHrd1 as indicated. Cells extract were prepared in RIPA buffer (50 mM Tris-HCl pH 7.5, 150 mM NaCl, 1% Sodium deoxycholate, 0.5% SDS, 0.5% NP-40, 1 mM EDTA) with the addition of protease inhibitors tablet (Roche Applied Science) and 0.1 mM PMSF. Extracts were mixed with beads conjugated with indicated antibodies at 4°C for 2–4 h. Immunoprecipitates were resolved by SDS-PAGE, transferred to PVDF membrane, and probed with antibodies as indicated.

### Detection of ubiquitylated ugPrP

Cells plated in 100-mm plates were transfected with the plasmid expressing ugPrP, and later treated with 50 µM MG132 for 6 h before the samples were collected. Cells were harvested at 48 h after transfection and lysed in RIPA buffer and then mixed with rProtein A beads coated with PrP antibody 3F4 at 4°C. The bound proteins were analyzed by western blotting with Ub antibody FK2 (Enzo Life Sciences, Farmingdale, NY) and PrP antibody.

## Supporting Information

File S1 **Supporting Figures.** Figure S1. ugPrP binds gp78 but not HsHrd1. (A) gp78 immunoprecipitates ugPrP. Plsamids expressing myc-tagged gp78 and/or ugPrP as indicated were transfected into HEK293 cells. Proteins were extracted and immunoprecipitated with beads coated with myc antibody. Immunoprecipitates were separated on SDS-PAGE, and probed with anti- 3F4 (top panel) or myc antibody (middle panel). The amounts of ugPrP in cell extracts were evaluated and presented in lower panel. (B) ugPrP does not immunoprecipitate HsHrd1. Proteins were extracted from cells expressing myc-tagged HsHrd1 and ugPrP. The indicated immunoprecipitations and immunoblottings were carried out as described above in (A). Figure S2. Quantitation of the data in Figure 3B. The experiments were done at least three times, and the average values with standard deviation are shown. Figure S3. Quantitation of the data shown in Figure 4B. The stability measurements were done more than three times, and the average values with standard deviation are shown.
(PDF)
