# Supplementary material for: Ubiquitin Ligase gp78 Targets Unglycosylated Prion Protein PrP for Ubiquitylation and Degradation
Source: PLoS One. 2014 Apr 8;9(4):e92290. doi: 10.1371/journal.pone.0092290 (PMC3979651; doi:10.1371/journal.pone.0092290)
Supplement: File S1 — Supporting Figures. Figure S1. ugPrP binds gp78 but not HsHrd1. (A) gp78 immunoprecipitates ugPrP. Plsamids expressing myc-tagged gp78 and/or ugPrP as indicated were transfected into HEK293 cells. Proteins were extracted and immunoprecipitated with beads coated with myc antibody. Immunoprecipitates were separated on SDS-PAGE, and probed with anti- 3F4 (top panel) or myc antibody (middle panel). The amounts of ugPrP in cell extracts were evaluated and presented in lower panel. (B) ugPrP does not immunoprecipitate HsHrd1. Proteins were extracted from cells expressing myc-tagged HsHrd1 and ugPrP. The indicated immunoprecipitations and immunoblottings were carried out as described above in (A). Figure S2. Quantitation of the data in Figure 3B. The experiments were done at least three times, and the average values with standard deviation are shown. Figure S3. Quantitation of the data shown in Figure 4B. The stability measurements were done more than three times, and the average values with standard deviation are shown. (PDF) [file pone.0092290.s001.pdf]

Supporting Figures

Figure S1. **ugPrP binds gp78 but not HsHrd1. (A)** gp78 immunoprecipitates ugPrP. Plsamids expressing myc-tagged gp78 and/or ugPrP as indicated were transfected into HEK293 cells. Proteins were extracted and immunoprecipitated with beads coated with myc antibody. Immunoprecipitates were separated on SDS-PAGE, and probed with anti-3F4 (top panel) or myc antibody (middle panel). The amounts of ugPrP in cell extracts were evaluated and presented in lower panel. **(B)** ugPrP does not immunoprecipitate HsHrd1. Proteins were extracted from cells expressing myc-tagged HsHrd1 and ugPrP. The indicated immunoprecipitations and immunoblottings were carried out as described above in (A).

Figure S2. **Quantitation of the data in Figure 3B.** The experiments were done at least three times, and the average values with standard deviation are shown.

Figure S3. Quantitation of the data shown in Figure 4B. The stability measurements were done more than three times, and the average values with standard deviation are shown.

Figure S1

A

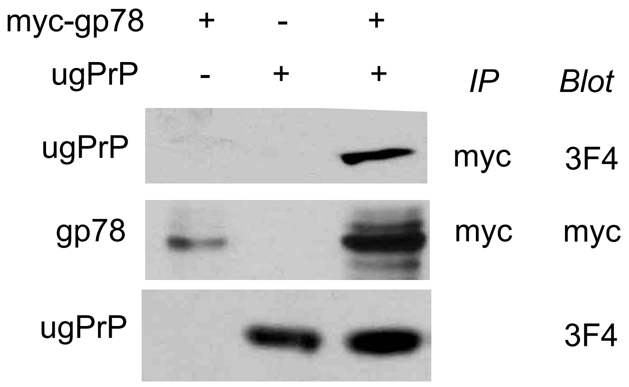
myc-gp78 + - +
ugPrP - + +
IP
Blot
ugPrP
myc
3F4
gp78
myc
myc
ugPrP
3F4

B

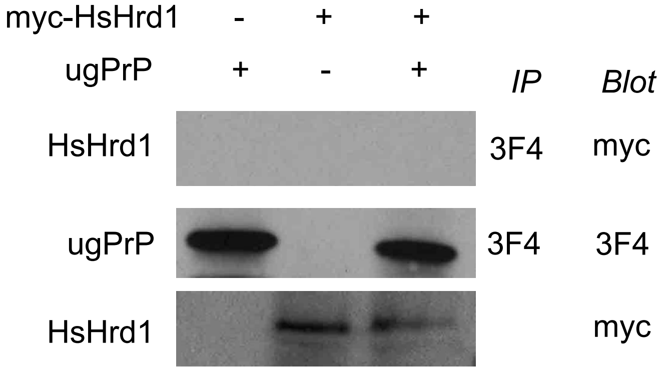
myc-HsHrd1 - + +
ugPrP + - +
IP
Blot
HsHrd1
3F4
myc
ugPrP
3F4
3F4
HsHrd1
myc

Figure S2

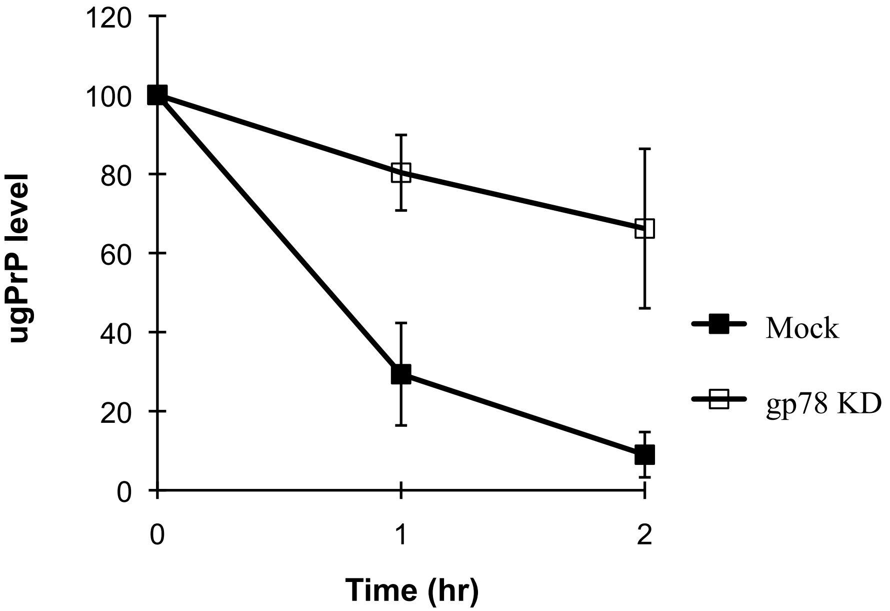

Figure S3

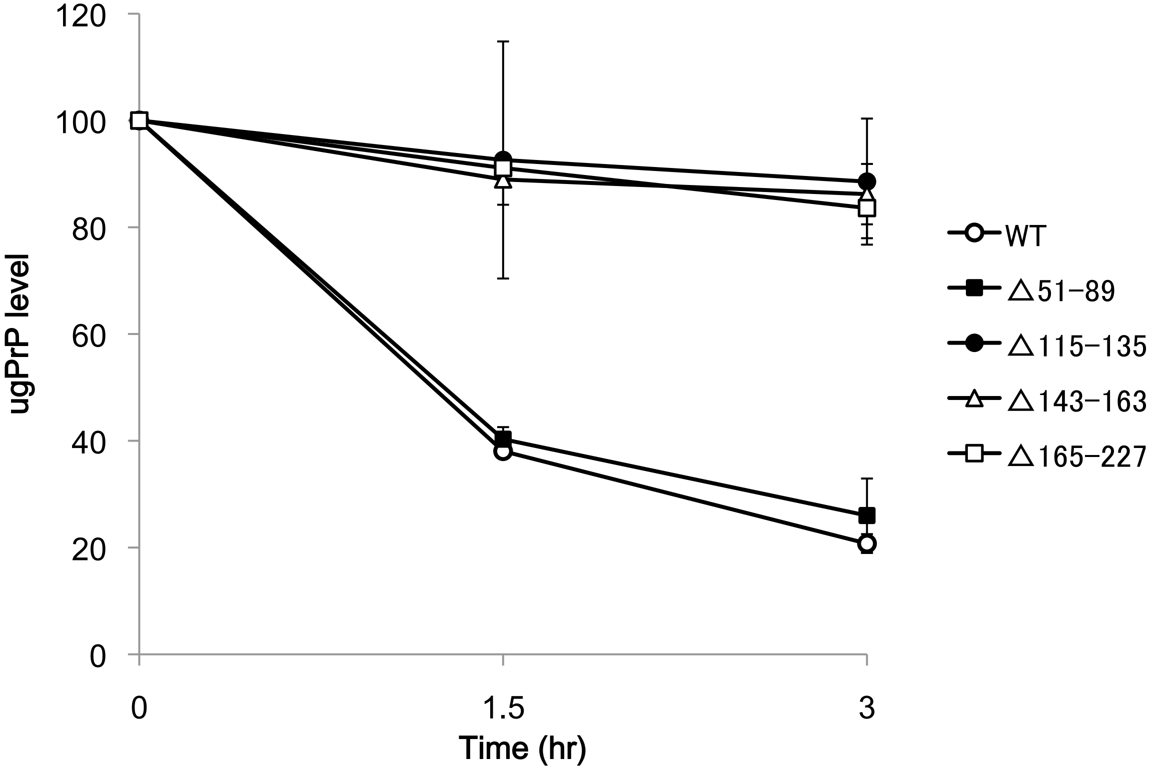
120
100
80
60
40
20
0
ugPrP level
0
1.5
3
Time (hr)
WT
△51-89
△115-135
△143-163
△165-227
